# Role of Endocytosis Proteins in Gefitinib-Mediated EGFR Internalisation in Glioma Cells

**DOI:** 10.3390/cells10113258

**Published:** 2021-11-21

**Authors:** Elisabete Cruz Da Silva, Laurence Choulier, Jessica Thevenard-Devy, Christophe Schneider, Philippe Carl, Philippe Rondé, Stéphane Dedieu, Maxime Lehmann

**Affiliations:** 1 UMR CNRS 7021, Laboratory of Bioimaging and Pathologies, University of Strasbourg, 67400 Illkirch, France; eslisasilva@gmail.com (E.C.D.S.); laurence.choulier@unistra.fr (L.C.); philippe.carl@unistra.fr (P.C.); philippe.ronde@unistra.fr (P.R.); 2 UMR CNRS 7369, Matrice Extracellulaire et Dynamique Cellulaire (MEDyC), Université de Reims Champagne Ardenne (URCA), 51687 Reims, France; jessica.devy@univ-reims.fr (J.T.-D.); christophe.schneider@univ-reims.fr (C.S.); stephane.dedieu@univ-reims.fr (S.D.)

**Keywords:** glioblastoma, cell invasion, membrane trafficking, tyrosine kinase inhibitors

## Abstract

EGFR (epidermal growth factor receptor), a member of the ErbB tyrosine kinase receptor family, is a clinical therapeutic target in numerous solid tumours. EGFR overexpression in glioblastoma (GBM) drives cell invasion and tumour progression. However, clinical trials were disappointing, and a molecular basis to explain these poor results is still missing. EGFR endocytosis and membrane trafficking, which tightly regulate EGFR oncosignaling, are often dysregulated in glioma. In a previous work, we showed that EGFR tyrosine kinase inhibitors, such as gefitinib, lead to enhanced EGFR endocytosis into fused early endosomes. Here, using pharmacological inhibitors, siRNA-mediated silencing, or expression of mutant proteins, we showed that dynamin 2 (DNM2), the small GTPase Rab5 and the endocytosis receptor LDL receptor-related protein 1 (LRP-1), contribute significantly to gefitinib-mediated EGFR endocytosis in glioma cells. Importantly, we showed that inhibition of DNM2 or LRP-1 also decreased glioma cell responsiveness to gefitinib during cell evasion from tumour spheroids. By highlighting the contribution of endocytosis proteins in the activity of gefitinib on glioma cells, this study suggests that endocytosis and membrane trafficking might be an attractive therapeutic target to improve GBM treatment.

## 1. Introduction

EGFR (epidermal growth factor receptor), a member of the ErbB tyrosine kinase receptor family, is commonly found amplified and/or mutated in near 60% of glioblastoma (GBM), the most aggressive brain tumour. In GBM, activated EGFR promotes PI3K/Akt (phosphatidyl-inositol-Kinase/Akt), MAPK/ERK (mitogen-activated protein kinases/extracellular signal-regulated kinases), signal transducer and activator of transcription 3 (STAT3), and phospholipase C gamma signalling cascades. These EGFR transduced signals promote GBM cell proliferation and invasion, and tumour progression [1,2].

EGFR signalling function is tightly regulated by endocytosis and membrane trafficking. Physiological EGFR endocytosis can occur through different pathways such as the clathrin-mediated endocytosis and non-clathrin endocytic pathway, depending on the nature and concentration of the ligand. Upon vesicle formation, dynamin-2 (DNM2), a GTPase protein, is recruited to pitch the vesicle from the plasma membrane [3,4], giving rise to the early endosomes (EE). In the EE, EGFR fate is decided, and the receptor is either transported to lysosomes for degradation or recycled back to the plasma membrane [5]. A critical group of endocytic regulators are the Ras-associated binding (Rab) proteins. In EE, Rab5 is responsible for cargo entry from the plasma membrane to the EE, generation of phosphotidylinositol-3-phosphate (PtdIns(3)P) lipid, homotypic fusion and actin/microtubules motility of EE and activation of endosomal signalling pathways [6].

In GBM, altered expression of EGFR membrane trafficking regulators, resulting in aberrant EGFR localisation, has been associated with tumour progression and therapy resistance to EGFR-targeted therapies [7,8,9,10,11]. Dysregulation of EGFR trafficking also occurs upon receptor mutation. For instance, *EGFRvIII*, the most common EGFR mutant in GBM, is inefficiently degraded as a consequence of either a high rate of recycling to the plasma membrane [12] or its translocation to the mitochondria wherein it triggers resistance to apoptosis [13].

Other studies have shown that EGFR trafficking is altered during therapeutic interventions and enlighten that this process may have important impact on patient therapeutic responses [14]. Compared to the physiological situation, under therapeutic stress, EGFR follows distinct endocytosis and trafficking routes in a ligand- and tyrosine kinase-independent way [15,16]. For instance, in vitro studies indicate that X-ray irradiation of human bronchial carcinoma cells promotes caveolin1-mediated EGFR internalisation, in a Src (proto-oncogene tyrosine-protein kinase) kinase-dependent way. After being internalised, EGFR is transported to the nucleus where it activates DNA-PK (deoxyribonucleic acid-dependent protein kinase) phosphorylation and enhances double strand breaks repair [17]. Moreover, cisplatin treatment induces EGFR endocytosis and its accumulation into multivesicular bodies (MVB), through the activation of the stress-induced p38-MAPK pathway [14,16,18]. In MVB, EGFR accumulation activates the ERK pathway to delay apoptosis and promote chemoresistance [16]. Additionally, EGFR-targeting antibodies used in clinic or ongoing clinical development are able to induce EGFR internalisation [19,20,21]. Finally, it has been shown that EGFR-targeting tyrosine kinase inhibitors (TKIs) also disturb EGFR trafficking in GBM cells and in various other cancer cell types. TKI can trigger EGFR translocation, in the autophagy compartment [14], in mitochondria [13] or in nucleuses [17].

Dysregulation of EGFR trafficking plays an essential role in cancer progression and response to anti-EGFR therapies. In a previous work, we showed that gefitinib and others TKI enhance EGFR endocytosis and EGFR accumulation in fused early endosomes [22]. The aim of the present work was to identify key proteins that contribute to gefitinib-mediated EGFR endocytosis. In the present study, we identified the contribution of three endocytic proteins DNM2, Rab5 and the LDL receptor-related protein 1 (LRP-1) in this process. Importantly, inhibiting endocytosis by targeting DNM2 or LRP-1 protects glioma cells against gefitinib treatment during cell dissemination from tumour spheroids. The present study enlightens us on the importance of endocytic proteins in gefitinib’s anti-tumoral effects on glioma cells.

## 2. Materials and Methods

### 2.1. Antibodies

The following antibodies were used for immunostaining. Anti-EGFR antibodies (clone D38B1) were from Cell Signaling (Danvers, MA, USA). Anti–EEA1 (clone 14/EEA1) was from BD Transductions (Allschwill, Switzerland). Antibody against LRP-1 (clone 8G1) was from Genetex (Irvine, CA, USA). Fluorescently labelled secondary antibodies were purchased from Invitrogen (Carlsbad, CA, USA) (Alexa Fluor−488; −568; −647). DAPI was purchased from Santa Cruz Biotechnology (Dallas, TX, USA). The following antibodies were used for immunoblotting: anti-EGFR antibody (D38B1) were from Cell Signaling (Danvers, MA, USA), anti-LRP-1 (EPR3724) from Abcam (Cambridge, UK), anti-DNM2 (G-4) and anti-Rab5 (D-11) were from Santa Cruz Biotechnology (Dallas, TX, USA) and anti-GAPDH (clone C65) from Millipore (Darmtadt, Germany). HRP-conjugated secondary antibodies were purchased from Invitrogen (Carlsbad, CA, USA). All other reagents were of molecular biology quality.

### 2.2. Cell Culture

The human glioblastoma cell line U87MG was obtained from ATCC (American Tissue Culture Collection, Rockville, MD, USA); T98G cells were from ECACC (European Collection of Authenticated Cell Cultures, Sigma-Aldritch, Hamburg, Germany). LN443 cells were kindly provided by Prof. Monika Hegi (Lausanne, Switzerland). According to the canSar database, these cell lines express wild-type EGFR [23]. GBM cells were maintained in Eagle’s minimum essential medium (EMEM) (Lonza, Verviers, Belgium) supplemented with 10% foetal bovine serum (FBS) (Dominique Dutscher, Brumath, France), 1% sodium pyruvate (Lonza, Verviers, Belgium) and 1% nonessential amino acid (Lonza, Verviers, Belgium), in a 37 °C humidified incubator with 5% CO_2_. 

### 2.3. Plasmid Transfection

YFP-Rab5 (kindly provided Dr. Marino Zerial (MaxPlanck Institut, Germany)), GFP-Rab5S324N (#35141) and GFP-Rab5Q79L (#35140) were from Addgene (Watertown, MA, USA). For siRNa mediated silencing experiments, we used siGENOME^TM^ siRNA smartpools (4 different siRNAs/pool): non-targeting siRNA pools (Dharmacon D-001206-14-05), siRNA-DNM2 (Dharmacon M-004007-03-0005), siRNA-LRP-1 (Dharmacon M-004721-01-0005) were used. A total of 0.25 × 10^6^ cells were used for each transient transfection using 1.5 µg for expression plasmid or 50 nM for siRNA using JetPrime^®^ (PolyPlus-Transfection, Illkirch, France) following the manufacturer’s instructions. Fusion protein expression was confirmed by fluorescent microscopy the day after, and downregulation of DNM2 or LRP-1 was assessed by immunoblotting 72 h after siRNA transfection.

### 2.4. Fluorescent Quantification of EGFR Endocytosis

EGF coupled to Alexa Fluor 488 (Molecular Probes, Invitrogen, Carlsbad, CA, USA) was used to study the ligand-induced EGFR endocytosis. For EGF uptake, cells were plated on coverslips previously coated with collagen-I (20 µg. mL^−1^ in DPBS) (Advanced BioMatrix, Carlsbad, CA, USA). Cells were serum-starved for 1 h at 37 °C, then washed in ice-cold DPBS and incubated on ice in serum-free culture medium containing 100 ng/mL Alexa Fluor 488–EGF. After incubation on ice for 30 min, cells were briefly washed with ice-cold DPBS. Cells fixed at this step were used as the negative control. Otherwise, cells were incubated with pre-warmed complete medium at 37 °C for 3 h in the presence of 20 µM gefitinib, an EGFR tyrosine kinase inhibitor (ChemiTek, Indianapolis, IN, USA) and pharmacological inhibitors of DNM2 (dynasore and dyngo-4a, ChemiTek, Indianapolis, IN, USA) or of LRP-1 (RAP), as indicated. His-tagged RAP was purified by gravity-flow chromatography using a nickel-charged resin as described previously [24]. Non-internalised EGF was stripped by incubating the cells with a solution of sodium acetate 0.2 M pH 2.7 for 5 min on ice. After washing, cells were fixed in 3.7% (*v*/*v*) paraformaldehyde (Electron Microscopy Sciences, Hatfield, PA, USA) for 8 min and stained with DAPI. Images were acquired using a confocal microscope (LEICA TCS SPE II, 60× magnification oil-immersion) (Leica, Nanterre, France). Analysis were performed after a threshold (identical for all conditions) was applied to eliminate background. The integrated fluorescence intensity of Alexa Fluor 488-EGF was determined in each cell. Image analysis was performed using ImageJ on at least 20 cells/experiments on 3 independent experiments.

### 2.5. Cell Surface Biotinylation and Endocytosis Assays

Subconfluent cells were placed at 4 °C to prevent internalisation, washed twice with ice-cold Hank’s Balanced Salt Solution (Gibco, Thermo Fischer Scientific, Braunsweig, Germany) containing 0.5 mM MgCl_2_ and 1.26 mM CaCl_2_ (Ca/Mg-HBSS) adjusted to pH 8, then incubated for 30 min with 1 mg·mL^−1^ EZ-Link Sulfo-NHS-LC-Biotin (Thermo Fisher Scientific, Braunsweig, Germany) in Ca/Mg-HBSS. After washing with ice-cold Ca/Mg-HBSS, free biotin was quenched with 20 mM glycine in Ca/Mg-HBSS. Following cell-surface biotinylation, cells were incubated 2 h at 37 °C in complete medium (w/wo gefitinib and/or RAP), to allow endocytosis. Cells were quickly replaced on ice, washed thrice with ice-cold Ca/Mg-HBSS, then washed twice to remove biotin from cell-surface proteins with 300 mM MesNa (Sigma-Aldrich, Hamburg, Germany) in buffer composed of Tris 50 mM pH 8,6, NaCl 100 mM, EDTA 1 mM, BSA 0.2% (Sigma-Aldrich, Hamburg, Germany). Cells were rinsed twice with Ca/Mg-HBSS, incubated with iodoacetamide (5 mg/mL) (Sigma-Aldrich, Hamburg, Germany) in Ca/Mg-HBSS, then washed with Ca/Mg-HBSS. To determine the total amount of surface biotinylation and to serve as a control, dishes were kept on ice after biotin labelling and protected from MesNa treatment. Whole-cells extracts were prepared, and biotinylated proteins were recovered from 100 µg of cell lysate by using avidin protein immobilised on agarose beads (Thermo Fisher Scientific, Braunsweig, Germany), subjected to SDS-PAGE, and revealed by immunoblotting with anti-EGFR.

### 2.6. Immunoblotting

For protein expression analysis (Figure S1), after 3 washes in ice cold PBS, proteins were extract using RIPA buffer (Thermo Fisher Scientific, Braunsweig, Germany), according to manufacturer protocol, protein concentrations were evaluated using DC Protein assay (Bio-Rad, Hercules, CA, USA) and 20 µg of proteins were further analysed. For siRNA experiments, proteins were directly extracted in Laemmli sample buffer (Bio-Rad, Hercules, CA, USA). Proteins were separated on precast gradient 4–20% SDS-PAGE gels (Bio-Rad, Hercules, CA, USA) and transferred to a PVDF membrane (GE Healthcare, Dornstadt, Germany). Membranes were probed with primary antibodies: anti-EGFR antibody, anti-DNM2, anti-Rab5 and anti-LRP-1 at 1µg/mL and anti-GAPDH at 0.2 µg/mL in blocking solution (TBS—tween 0.1%, 5% non-fat dry milk). Immunological complexes were revealed with anti-rabbit or anti-mouse IgG coupled peroxidase antibodies using chemoluminescence (ECL detection reagent, GE Healthcare, Dornstadt, Germany) and visualised with a LAS4000 image analyser (GE Healthcare, Dornstadt, Germany). GAPDH was used as the loading control for all samples.

### 2.7. Rab5 Activation Assay

Upon gefitinib treatment, active Rab5 immunoprecipitation was performed using a conformation specific anti-active Rab5 antibody following the manufacturer’s instructions (NewEast Biosciences, King of Prussia, PA, USA). Rab5 protein in the total extract and in the immunoprecipitate was revealed using anti-Rab5 (D11) antibody by immunoblotting.

### 2.8. Confocal Microscopy and Image Analysis

Coverslips were coated with collagen-I (20 µg/mL in DPBS). A total of 20,000 cells were seeded in serum containing medium and cultured for twenty-four hours before gefitinib treatment. Alternatively, two-day-old spheroids were seeded in complete medium and treated with 20 µM of gefitinib. Cells were fixed in 3.7% (*v*/*v*) paraformaldehyde during 20 min, permeabilised with 0.1% Triton-X100 (Euromedex, Souffelweyrsheim, France) for 5 min. After a 3 h blocking step using PBS-BSA 3% solution, cells were incubated with primary antibodies O/N at 4 °C (2 µg/mL each in PBS-BSA 3%). Cells were rinsed in PBS 1X and incubated with appropriate secondary antibodies (1 µg/mL in PBS-BSA 3%) and DAPI for 1 h. Samples were mounted on microscope slides using a fluorescence mounting medium (Dako, Carpinteria, CA, USA). Images were acquired using a confocal microscope (LEICA TCS SPE II, 60× magnification oil-immersion) (Leica, Nanterre, France). For each experiment, identical background subtraction was applied to all images. Pearson correlation coefficients from 8 images (2–4 cells per images) from 3 independent experiments were calculated using Colocalization_Finder ImageJ software. The 3D reconstructions corresponding to confocal images Z-stacks obtained using stacks of 350 nm. The 3D image reconstruction was performed using IMARIS software.

### 2.9. Spheroid Migration Assays

Methylcellulose solution was made as previously described [25]. A single cell suspension was mixed in EMEM/10%FBS containing 10% of methylcellulose. All the spheroids were made with 1000 cells by hanging drop methods in a 20 μL drop [25]. Tissue culture plastic dishes were previously coated with 10 µg·ml^−1^ of collagen-I in DPBS solution for 2 h at 37 °C. Two-day-old spheroids were allowed to adhere and migrate in complete medium (EMEM, 10% FBS). Twenty-four hours later, cells were fixed with paraformaldehyde 3.7% and nuclei were stained with DAPI. Nuclei were picturised under the 5x objective in the fluorescence microscope ZEISS-Axio (ZEISS, Oberkochen, Germany). Image analysis to evaluate the number of cells that migrated out of the spheroid was performed with ImageJ software using a homemade plugin.

### 2.10. Statistical Analysis

Statistical analysis between samples was conducted by one-way analysis of variance (ANOVA) followed by a Bonferroni post-test with the GraphPad Prism program, unless otherwise stated. The significance level is controlled by 95% confidence interval.

## 3. Results

### 3.1. Knock-Down of DNM2 Decreases Gefitinib-Mediated EGF Endocytosis

**Figure 1:**
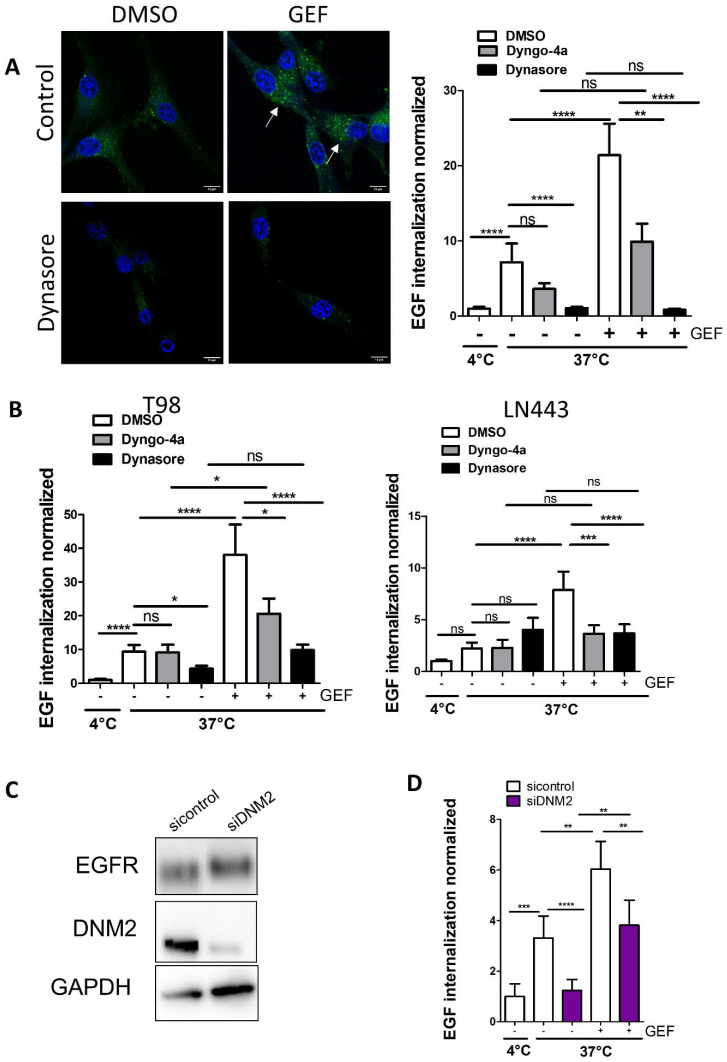
Gefitinib-mediated EGF internalisation is dependent on DNM2. EGF-internalisation assays were performed in the presence gefitinib (20 µM) for 1 h. (**A**,**B**) DNM2 GTPase activity was inhibited by treatment with either dynasore (12 µM) or dyngo-4A (10 µM) (**A**) Left panel: confocal images of control and dynasore-treated cells, showing, in green, internalised EGF-Alexa Fluor 488 upon incubation at 37 °C. Arrows show internalised EGF. Scale bar = 12 µm. Right panel: the internalisation was quantified by integrated fluorescence density on 20 cells of 3 independent experiments. Data reported as column histograms are the mean with 95% CI. (**B**) Results were confirmed in other GBM cell lines. EGF-internalisation assays were performed in T98 and LN443 cells using dynasore and dyngo-4A as described in A. (**C**) Downregulation of DNM2 expression was obtained by siRNA-DNM2 mediated silencing. DNM2 silencing was confirmed by immunoblotting after 72 h. EGFR protein was also immunodetected and remained constant in both conditions. GAPDH was used as the loading control. (**D**) EGF-internalisation assays were performed on U87 cells transfected with siRNA-control or U87 siRNA-DNM2. * *p* < 0.5; ** *p* < 0.01; *** *p* < 0.001; **** *p* < 0.0001; ns: not significant.

We have previously shown that in, U87, T98G and LN443 GBM cells that express wild type EGFR [23], cytostatic concentrations of gefitinib lead to the accumulation of EGFR in enlarged early endosomes and increase EGF endocytosis, a phenomenon we called gefitinib-mediated endocytosis (GME) [22]. To better characterise the molecular mechanisms underlying GME, we first seek to determine the potential involvement of DNM2, which is critical in physiological ligand-dependant EGFR endocytosis [26]. As shown by immunoblot experiments, EGFR and DNM2 expression was first compared between three GBM cell lines used in this study. We also showed that gefitinib treatment had little impact on their expression level (Figure S1). We first examined the effect of dynasore and dyngo-4A, two potent pharmacological inhibitors of DNM2 GTPase activity [27,28], on Alexa Fluor 488 EGF endocytosis in U87 cells. The off-target effect was shown at concentrations of 80 µM for dynasore and 30 µM for dyngo-4A [29]. To reduce this potential risk, we selected concentrations of 12 µM and 10 µM for dynasore and dyngo-4A, respectively. As shown by confocal images and fluorescence quantification, dynasore was able to inhibit physiological EGF endocytosis by 86%, which is in agreement with the established role of DNM2 in ligand-mediated EGFR endocytosis [26]. Dyngo-4A had the tendency to inhibit EGF endocytosis although with no significant differences compared to DMSO-treated cells. As previously described [22], compared to physiological untreated conditions, gefitinib addition in the culture medium of U87 cells increased EGF endocytosis two-fold (Figure 1A). Interestingly, dynamin inhibitors significantly inhibited GME of EGF (96% for dynasore and 53% for dyngo-4A) (Figure 1A). Although dynasore but not dyngo-4A inhibited the physiological endocytosis of EGF in T98 cells and no drug affected that in LN443 cells, the two drugs potently inhibited GME of EGF in both cell lines. Of note, we observed some increase in EGF endocytosis in Dyngo-4A+GEF vs. Dyngo-4A+DMSO in U87 cells (although non-significant) and T98 cells, which may reflect that dyngo-4A was less potent than dynasore in inhibiting GME (Figure 1B). To further confirm the involvement of DNM2 in GME of EGF, we silenced *DNM2* expression in U87 cells using a pool of siRNA. SiRNA-DNM2 efficiently repressed DNM2 expression and had no impact on EGFR expression (Figure 1C). DNM2 downregulation inhibited physiological Alexa Fluor 488 EGF endocytosis, and more importantly dampened EGF internalisation in gefitinib-treated cells (Figure 1D). Together these results confirmed the role of DNM2 in GME.

### 3.2. Gefitinib Activates Rab5 to Promote EGFR Endocytosis

**Figure 2:**
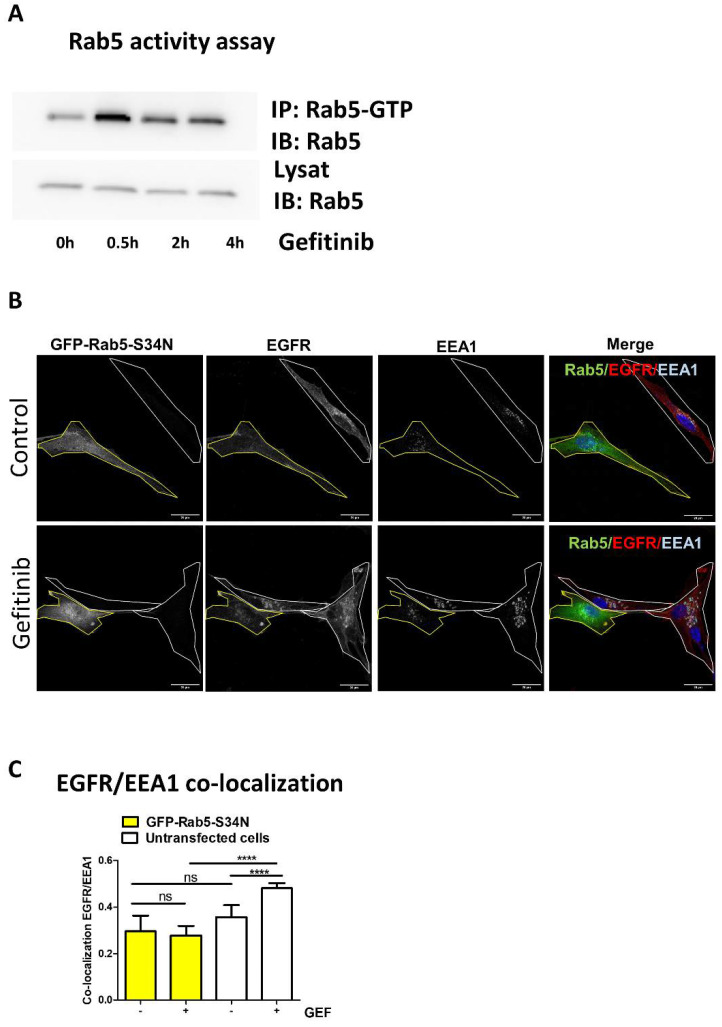
Gefitinib activates Rab5 to promote EGFR endocytosis. (**A**) Rab5 activity assay was performed in cell lysates upon gefitinib treatment. U87 cells were seeded the day before gefitinib treatment (20 µM) for different periods of time. Cells were lysed and Rab5-GTP was immunoprecipitated. Rab5 was immunodetected in IP and control lysates. (**B**) U87 cells seeded on glass coverslips were transiently transfected with a dominant negative Rab5 mutant (GFP-Rab5-S34N). After treatment with gefitinib (4 h, 20 µM), cells were fixed, then EGFR and EEA1 were immunodetected and analysed by confocal imaging. Single or merged channel images are represented. Transfected cells are delimited in yellow in all images. Scale bar = 20 μm. (**C**) EGFR/EEA1 co-localisation upon gefitinib treatment in each untransfected cell (white bars) and GFP-Rab5-S34N expressing cell (yellow bars) was evaluated using Pearson’s correlation coefficient from 8 images (2–4 cells per images) in 3 independent experiments. Data reported as column histograms are the mean with 95% CI. **** *p* < 0.0001; ns: not significant.

The monomeric GTPase Rab5 plays an essential function in EGFR endocytosis [30,31,32]. Notably, overactivation of Rab5 leads to EGFR endocytosis and its accumulation in large, fused endosomes [32,33,34]. In the first few hours of gefitinib treatment, GME is also characterised by the formation of enlarged early endosomes that accumulate EGFR [22]. Thus, we seek to determine the potential role of Rab5 in GME. First, we transiently expressed a recombinant wild-type Rab5 (YFP-Rab5) or a constitutively active Rab5 mutant (GFP-Rab5-Q79L) in U87 cells. In line with data from Ceresa’s studies [32], we observed that overexpression of GFP-Rab5-Q79L, and to a lesser extent YFP-Rab5, triggered an accumulation of EGFR into enlarged early endosome antigen 1 (EEA1)-positive vesicles (Figure S2A). Quantification of EGFR/EEA1 co-localisation was performed in each cell expressing or not expressing GFP-Rab5-Q79L (Figure S2B). As expected, EGFR/EEA1 co-localisation was increased in cells expressing constitutively active Rab5 compared to non-expressing cells. This indicates that gefitinib might activate Rab5 to promote EGFR accumulation into early endosomes. To test this hypothesis, we immunoprecipitated the active GTP-bound Rab5 protein using a conformation sensitive anti-Rab5 mAb. Time course experiments revealed that gefitinib increased Rab5 activity upon 30 min of treatment and maintained Rab5 active for at least 4 h (Figure 2A). To confirm that Rab5 activation by gefitinib is necessary for GME, we quantified EGFR recruitment into early endosome in U87 cells that transiently expressed the dominant-negative (DN) Rab5 mutant (GFP-Rab5-S34N) compared to non-expressing cells (Figure 2B). By contrast, with untransfected cells, in GFP-Rab5-S34N expressing cells, EGFR was barely found in EEA1-positive endosomes after gefitinib treatment (Figure 2B,C). Altogether, these results support that gefitinib activated Rab5, by a still-unknown mechanism, to promote EGFR endocytosis, and enlighten that GME shares common features with physiological EGFR endocytosis.

### 3.3. EGFR Is Recruited into LRP1 Rich Endosomes upon Gefitinib Treatment

**Figure 3:**
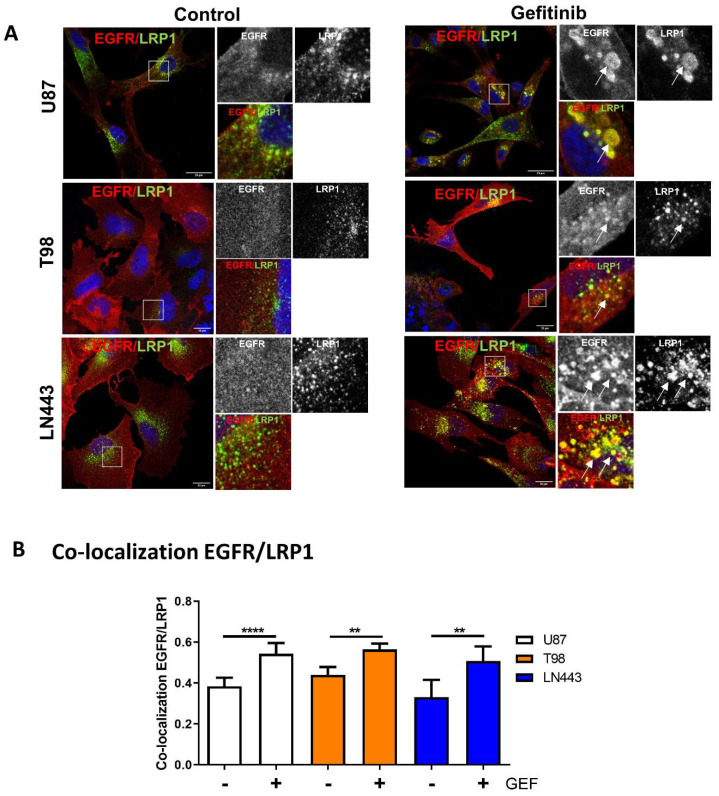
Gefitinib re-localises EGFR on LRP-1-positive endosomes. (**A**) U87, T98 and LN443 two-day-old spheroids were seeded on collagen-I-coated (20 µg·mL^−1^) glass coverslips. After 24 h of treatment with vehicle DMSO (Control) or 20 µM of gefitinib (Gefitinib), spheroids were fixed, EGFR (red) and LRP-1 (green) were immunodetected and analysed by confocal microscopy. Magnified images are from the inserts into the peri-nuclear area, either in single channels or in merged. Arrows indicate GME co-internalised EGFR and LRP-1. Scale bar = 20 μm. (**B**) EGFR/EEA1 co-localisation upon gefitinib treatment was determined using Pearson’s correlation coefficient from 8 images (2–4 cells per images) from 3 independent experiments. Data, reported as column histograms, are the mean with 95% CI ** *p* < 0.01, **** *p* < 0.0001.

**Figure 4:**
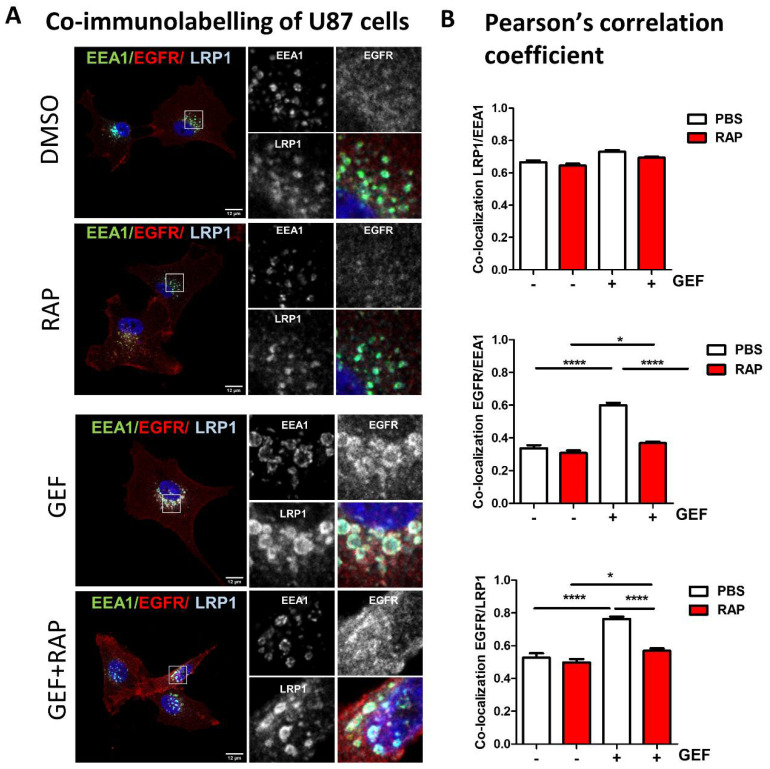
LRP-1 antagonist decreases EGFR/LRP-1 co-localisation into early endosomes. (**A**) U87 cells were seeded on glass coverslips. After 4 h of treatment with gefitinib (20 µM) and/or of RAP (500 nM), cells were fixed, EEA1 (green), EGFR (red) and LRP-1 (cyan) were immunodetected and analysed by confocal microscopy. Magnified images are from the inserts into the peri-nuclear area, either in single channels or in merged ones. Scale bar = 20 μm. (**B**) Co-localisation was determined using Pearson’s correlation coefficient from 40–60 cells from 3 independent experiments. Data, reported as column histograms, are the mean +/− SEM * *p* < 0.5, **** *p* < 0.0001.

Global endocytosis processes appear to be affected by gefitinib treatment, thus opening the possibility that, similar to Rab5 or DNM2, other endocytosis proteins may be involved in GME. The low-density lipoprotein receptor-related protein-1 (LRP-1), a large multifunctional receptor belonging to the low-density lipoprotein receptor family, controls the endocytosis of more than 30 different ligands including growth factor receptors. However, no functional interaction with EGFR has been established yet [35]. Data depicted in Figure S1 (immunoblot on cell lysate) indicate that LRP-1 was expressed at different levels in the three GBM cell lines studied and that its expression was not affected by gefitinib treatment. We analysed the impact of gefitinib on LRP-1 and EGFR localisation in cells that were treated for 24 h with gefitinib (Figure 3). Confocal imaging revealed that, upon gefitinib treatment, EGFR was detected in large LRP-1-positive endosomes (Figure 3A). We obtained similar results on LN443 cells and to a lesser extent on T98 cells (Figure 3A and Figure S1). In agreement with these observations, image analysis showed that gefitinib treatment significantly increased EGFR/LRP-1 co-localisation in the three GBM cell lines (Figure 3B). As shown in Figure 4, after gefitinib treatment both LRP1 and EGFR accumulated in EEA1- early endosomes. These data enlighten a potential link between EGFR and LRP1 during gefitinib-mediated endocytosis of EGFR in GBM cells.

### 3.4. LRP-1 Is Involved in Gefitinib-Mediated EGFR Endocytosis

**Figure 5:**
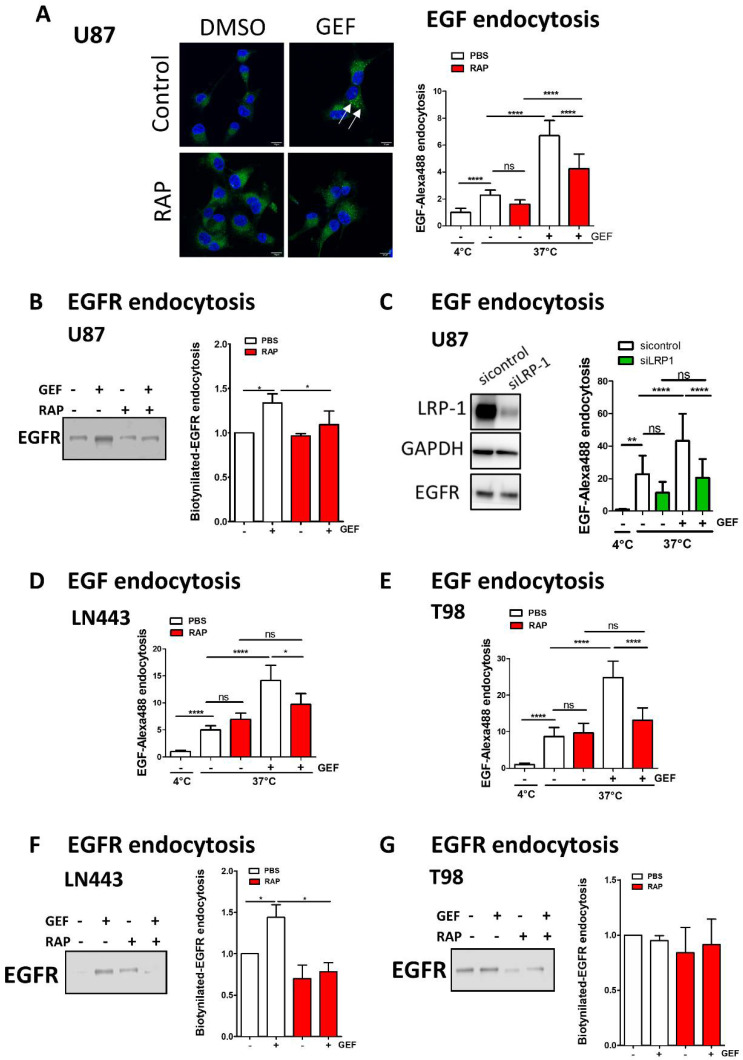
Inhibition of LRP-1 decreases GME. (**A**) EGF-Alexa 488 for EGF-internalisation assay were performed on U87cells in presence in the culture medium of gefitinib (20 µM) and/or of RAP (500 nM) for LRP-1 inhibition. Nucleuses were stained with DAPI. Left panel: confocal images showing in green EGF-Alexa Fluor 488 internalised upon incubation at 37 °C. Scale bar = 12 µm. Right panel: quantification of EGF-Alexa Fluor 488 fluorescence intensity was performed on 20 cells on 3 independent experiments. Data, reported as column histograms, are the mean +/− s.d. (**B**) Following cell-surface biotinylation, cells were incubated in complete media, with or without gefitinib (15 µM) and with or without RAP (500 nM) for 3 h. Cells were treated with MESNa agent to remove biotin present on cell-surface proteins. After purification, biotinylated proteins were then subjected to EGFR immunoblot. Top panel: quantification of EGFR protein bands (mean of 4 independent experiment). Lower panel: immunoblot showing the endocytosis of biotinylated EGFR. (**C**) Left panel: downregulation of LRP-1 expression was obtained by silencing using siRNA-LRP-1. EGF-internalisation assay was further performed on U87 transfected with siRNA-control (white bars) and siRNA-LRP-1 (green bars). Right panel: LRP-1 silencing was confirmed by immunoblotting 72 h after transfection. The EGFR protein level was controlled and remained constant in both conditions. GAPDH was used as the loading control. (**D**,**E**) EGF-internalisation assay was performed in LN443 (**D**) and T98 (**E**) cells as described in A. (**F**,**G**) EGFR-internalisation assay was performed in LN443 (**F**) and T98 (**G**) cells as described in B. Data, reported as column histograms, are the mean with 95% CI. * *p* < 0.5, ** *p* < 0.01; **** *p* < 0.0001; ns: not significant.

To determine whether LRP-1 may have any impact on gefitinib-mediated EGFR endocytosis, we first used recombinant protein RAP (receptor-associated protein) an endogenous LRP-1 molecular chaperone that antagonises LRP-1 binding to its ligands [36,37]. Confocal images of immunolabelled U87 cells revealed that RAP strongly reduced EGFR recruitment in the LRP-1-rich EEA1+-early endosome induced by gefitinib treatment (Figure 4A,B). Of note, neither gefitinib nor RAP impacted the co-localisation level between LRP1 and EEA1 (Figure 4B). To go further, we next examined the effect of RAP on Alexa Fluor 488-EGF endocytosis in U87 cells (Figure 5A). Confocal images of EGF endocytosis assays (Figure 5A- left panel) indicate that addition of RAP in the culture medium dampens the impact of gefitinib on EGF internalisation. Quantification of integrated fluorescence in individual cells showed that RAP had a limited, non-significant, impact on physiological EGF endocytosis but significantly inhibited gefitinib-mediated EGF endocytosis on U87 cells (Figure 5A-right panel). We then directly monitored EGFR endocytosis after cell surface biotinylation and showed that LRP-1 inhibition by RAP decreased gefitinib-mediated EGFR internalisation (Figure 5B). To better demonstrate the role of LRP-1 in GME, we next used siRNA-mediated silencing of LRP-1 in U87 cells. LRP-1 expression was efficiently downregulated by siRNA-LRP-1, while EGFR expression remained intact (Figure 5C). LRP-1 knockdown inhibited gefitinib-induced EGF internalisation to a similar extent compared to RAP treatment (Figure 5A,C). As observed on U87 cells, in LN443, inhibition of LRP-1 by RAP effectively reverses gefitinib stimulation of EGF and EGFR internalisation, but not their physiological endocytosis (Figure 5D,F). Concerning T98 cells, we obtained similar data using EGF endocytosis assay (Figure 5E); however, an experiment based on cell surface biotinylated-EGFR unexpectedly failed to reveal an increase in EGFR internalisation by gefitinib or its inhibition by RAP (Figure 5G). Although we observed cell-to-cell variation, our results highlight the contribution of LRP-1 to gefitinib-mediated EGFR endocytosis and shed light on the first ever described functional connection between LRP-1 and EGFR.

### 3.5. Endocytosis Is Critical for Gefitinib-Mediated Inhibition of GBM Cell Dissemination from 3D Spheroids

**Figure 6:**
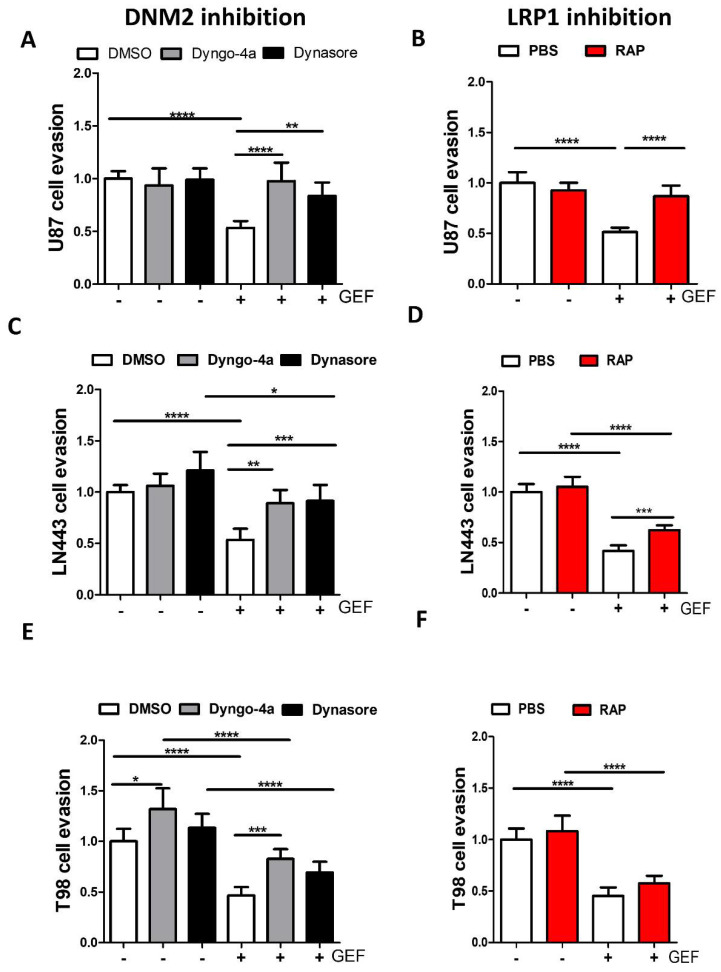
GME inhibition decreases gefitinib efficacy on cell evasion. (**A**) U87 two-day-old spheroids were plated onto collagen-I-coated (10 µg·mL^−1^) plastic dishes. Spheroids were treated with DNM2 GTPase activity inhibitors (10 µM of dyngo-4a or 12 µM of dynasore) and/or 20 µM of gefitinib for 24 h. After DAPI staining, the number of evading cells was quantified by automated counting of nuclei using an ImageJ homemade plugin. Data are represented in column histograms. (**B**) U87 two-day-old spheroids were plated as described above and treated with LRP-1 antagonist RAP (500 nM) and/or 20 µM of gefitinib. (**C**,**D**) Cell evasion assays from 3D tumour spheroids using dynasore or dyngo-4A (**C**) or RAP (**D**) were performed with T98 gefitinib-treated cells. (**E**,**F**) Cell evasion assays from 3D tumour spheroids using dynasore or dyngo-4A (**E**) or RAP (**F**) were performed with LN443 gefitinib-treated cells. * *p* < 0.5, ** *p* < 0.05 *** *p* < 0.001, **** *p* < 0.0001.

Endocytosis and membrane trafficking play important roles in tumour cell migration and invasion [38,39,40] and EGFR trafficking dysregulation has been associated with an invasive profile on glioma cells [8]. It thus appears important to determine whether GME may have an impact in gefitinib-mediated inhibition on glioma cell invasion. We previously showed, using cell evasion from tumour spheroids plated on a collagen-coated surface [25], that gefitinib reduced the number of U87 evading cells by almost 50% [22]. In a first series of experiments, the number of cells that migrate out of the spheroids was quantified in presence of dynasore or dyngo-4A (to inhibit DNM2) or RAP (to inhibit LRP-1) (Figure 6). In the absence of gefitinib, DNM2 inhibitors had no impact on the number of disseminated cells in control conditions but were able to restore efficient cell evasion in gefitinib-treated cells (Figure 6A). Similarly, LRP-1 inhibition by RAP increased the number of evading cells upon gefitinib treatment of U87 by 1.8-fold (Figure 6B). As observed on U87 cells, DNM2 inhibitors protected LN443 and T98 cells from gefitinib (Figure 6C–F). Results on LRP-1 inhibition show some cell-to-cell variations. RAP efficiently enhanced evasion of gefitinib-treated LN443 cells but had no impact on gefitinib-treated T98 cells.

**Figure 7:**
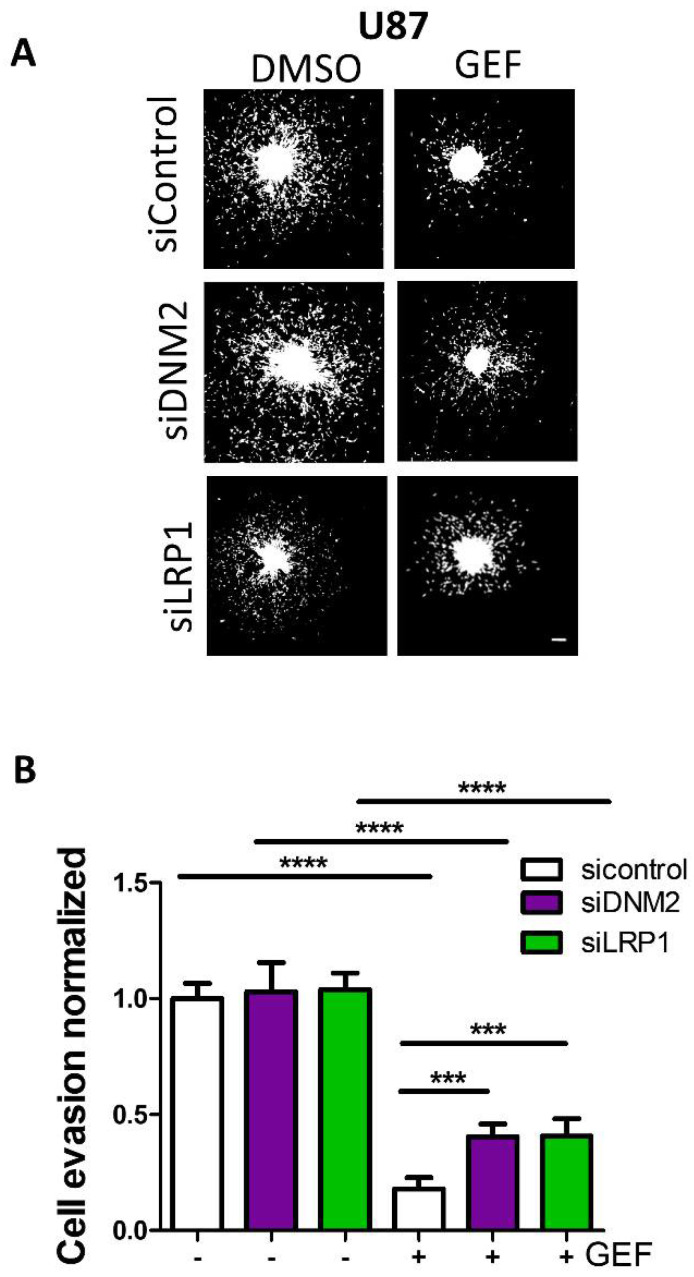
DNM2 or LRP-1 silencing decreases gefitinib efficacy on cell evasion. (**A**) U87 transfected with siRNA-control or siRNA targeting DNM2 or LRP-1 two-day-old spheroids were plated onto collagen-I-coated (10 µg·mL^−1^) plastic dishes. After 24 h of gefitinib treatment, spheroids were fixed, and nucleus were labelled by DAPI staining. Fluorescent microscopy images of representative spheroids after 24 h of migration were taken. Scale bar: 300 µm. (**B**) After DAPI staining, the number of evading cells was quantified by automated counting of nuclei using a previously validated ImageJ homemade plugin. Data are represented in column histograms. *** *p* < 0.001, **** *p* < 0.0001.

Finally, to confirm the protective role of DNM2 or LRP-1 inhibition, prior to spheroid formation, we transfected U87 cells with siRNA targeting either DMN2 or LRP-1. Figure 7A depicted fluorescent microscopy images of DAPI-labelled cells that escaped from a spheroid 24 h after seeding on a collagen-coated substratum. It can be observed that neither siRNA-DNM2 nor siRNA-LRP-1 had noticeable impact on the capability of the cell to escape from the spheroid. Quantification showed that gefitinib inhibited the number of evading cells in siRNA-control transfected cells by 82% and cell evasion of DNM2 or LRP-1 knockdown cells by approximately 60%. Importantly, both silencing of DNM2 and LRP-1 significantly increased more than two-fold the cell evasion of gefitinib-treated spheroids (Figure 7B). In conclusion, we identified two endocytosis proteins involved in GME whose expression level and function participated in GBM cell response to gefitinib treatment.

## 4. Discussion

We recently showed that, in various GBM cells, gefitinib perturbs membrane trafficking and increases EGFR endocytosis [22]. In the present in vitro study based on three different GBM cells, we identified three endocytosis proteins, DNM2, Rab5 and LRP-1 as key regulators of gefitinib-mediated EGFR internalisation. Using pharmacological and siRNA-mediated approaches, we showed that DNM2 inhibition or downregulation efficiently counteracted gefitinib-mediated EGFR endocytosis. Furthermore, we showed that gefitinib treatment leads to Rab5 activation and that expression of a dominant-negative mutant form of Rab5 dampened GME of EGFR. Confocal images revealed that EGFR is localised in LRP-1-rich early endosomes upon gefitinib treatment. Functional inhibition and silencing experiments showed that LRP-1 was not involved in physiological EGFR endocytosis but played an important role in gefitinib-mediated EGFR endocytosis. Several studies have shown that changes in the level of expression of proteins regulating EGFR trafficking affect cancer cell sensitivity to targeted therapies [7,8,10]. Using cell dissemination from spheroids, we showed that inhibition of DNM2 or LRP-1 confers greater resistance to gefitinib. Our results reveal that endocytosis plays an unexpected role in gefitinib action and that expression level of endocytosis proteins such as DNM2, LRP-1 or Rab5 could be relevant biomarkers to predict TKI efficiency in limiting dissemination of GBM cells from tumour spheroids.

DNM2, a large GTPase protein in charge of the endocytic fusion of clathrin coated pits, has been shown to play a significant role in EGFR endocytosis [26]. Here, using pharmacological inhibitors dynasore and dyngo-4A and by siRNA-mediated silencing, we showed that DNM2 plays a significant role in GME of EGFR and that its inhibition increased the disseminating potential of gefitinib-treated GBM cells. The role of DNM2 in cancer cell migration and invasion is a matter of debate. Some reports indicate that DNM2 stimulates migration and invasion of cancer cells, including glioma cells [41,42,43,44]. DNM2 has been shown to activate RAC1 and lamellipodia formation [43], to stabilise F-actin and filopodia [45], and to promote the invadopodia invasive function [46]. Others have shown that DNM2 downregulation promotes EGFR signalling and cancer cell motility [46,47]. In our experimental setup, DNM2 inhibition or repression had no impact in evasion of controlled cells indicating that DNM2 may not play an important function in the capacity of GBM cells to detach from tumour spheroids and to migrate. These results also suggest that dynasore and dyngo-4A or siRNA-targeting DNM2 and LRP-1 increased the evasion of gefitinib-treated cells, most likely by blocking GME rather than by stimulating cell migration.

The contribution of Rab5 in GME of EGFR was highlighted by the activation of Rab5 by gefitinib treatment over a time course compatible with endocytosis stimulation, and by inhibition of GME EGFR in DN-Rab5 expressing cells. Moreover, gefitinib treatment phenocopy Rab5-Q79L expression, as characterised by a massive distribution of EGFR into fused early endosomes [33]. The role of Rab5 in glioma progression and resistance to anti-EGFR therapy is still a matter of debate. Indeed, a recent study reported that in humans, Rab5 is overexpressed in glioma tissue compared to normal brain and that overexpression of Rab5 leads to enhanced proliferation and migration, which can be reversed by knockout of Rab5 [48]. By contrast, it has been shown that Rab5 inhibition sustains aberrant oncogenic EGFR signalling. For instance, Golgi phosphoprotein 3 (GOLPH3), a protein implicated in multiple cellular functions, was reported to promote glioma progression by inhibiting Rab5-dependent EGFR endocytosis [49]. Conversely, the tumour suppressors CMTM3 and CMTM7 (chemokine-like factor-like MARVEL transmembrane domain-containing 3 and 7) inhibit EGFR-mediated tumorigenicity and EGFR-dependent cell migration by stimulating Rab5 activity, in gastric and lung carcinomas, respectively [50,51]. Further studies are therefore required to delineate the role of Rab5 in glioma progression. An important result is that gefitinib treatment led to Rab5 activation to increase EGFR endocytosis. Thus, in line with results obtained on DNM2 or LRP-1, we speculate that Rab5 inhibition would hamper gefitinib anti-invasive function. This possibility was indirectly investigated in two recent studies which reported conflicting results. GOLPH3 has been reported to enhance the anti-tumoral activity of gefitinib in GBM cell lines [10], suggesting that Rab5 inhibition would sensitise cells to gefitinib. By contrast, compared to monotherapies, co-delivery of siRNA targeting GOLPH3 and gefitinib in brain tumours reduces cancer progression and improves mice survival [52]. The molecular mechanism by which gefitinib may activate Rab5 has not be investigated yet. An attractive hypothesis would be that, similar to cisplatin, UV radiation or anisomycin, gefitinib may accelerate ligand-independent EGFR endocytosis by stimulating the stress-activated p38-MAPK (MAPK14) [16,18,53,54,55,56,57]. Several mechanisms have been proposed, such as stress-activated p38 being able to directly phosphorylate EGFR on Ser1015 in lung cancer cells. Alternatively, p38 has been shown to be a major regulator of Rab5 activity. P38 can either phosphorylate EEA1 and rabenosin, two effectors of Rab5 [58] or phosphorylate the GDP dissociation factor, which releases inactive Rab5-GDP from the endosomal membrane and allows the maintenance of Rab5 in the cytoplasm for its subsequent activation [53]. P38 was shown to promote EGFR endocytosis, and its pharmacological inhibition leads to sustained EGFR expression in glioma stem cells [59]. We thus speculate once more that targeting endocytosis by p38 inhibition would reduce Rab5-mediated EGFR endocytosis and increase glioma cell resistance to gefitinib as has been found in the case of cisplatin treatment of U87 cells [60].

Despite the growing interest of the scientific community in understanding LRP-1 functionalities in tumour progression and resistance to treatments, the role of LRP-1 in the regulation of EGFR trafficking and glioma cell resistance to TKI had never been addressed so far. Our results highlight for the first time the instrumental role of LRP-1 in gefitinib-mediated EGFR internalisation and glioma cell dissemination. Although the mechanistic features have yet to be further deciphered, it is likely that β1 integrin may constitute a cell-surface molecular relay. β1 integrin was indeed reported to promote the endocytic machinery of EGFR in cancer cells [61] while LRP-1 was identified as a trigger for β1-integrin intracellular trafficking in the tumour context [62]. Consistently, we recently demonstrated that the endosomal pathway of α5β1 dimer in glioblastoma overlaps that of EGFR in response to gefitinib [22]. Our results should lead to considering LRP-1 as one molecular component mediating gefitinib efficacy in reducing GBM cell dissemination and infiltration. It therefore seems critical to consider the LRP-1 expression level to refine clinical designs using TKI, especially because LRP-1 depletion is found in invasive tumour areas that are the most refractory to treatments [63].

Membrane trafficking is often deregulated in cancer and contributes significantly to the antitumor activity of gefitinib. Therefore, therapeutic manipulation of endocytosis may represent an interesting strategy to increase the potency of EGFR TKI. The present work and other studies [6,7,8,10] have shown that intensive endocytosis is associated with increased sensitivity of glioma cells to TKI treatment. Predictably, in vivo targeting of proteins inhibiting endocytosis such as GOLPH3 or the Na+/H+ exchanger NHE9 represent an attractive therapeutic strategy to limit EGFR oncogenic activity and to increase cancer cell responsiveness to TKI [8,52]. A milestone was achieved by Simpson groups, who recently revealed that tumours can be classified based on EGF endocytosis profile from an ex vivo EGF endocytosis assay to predict antibody-based anti-EGFR therapy efficacy [64,65]. In the end, the analysis of protein expression levels alone does not always provide sufficient information to predict the clinical benefits of a targeted therapy or to stratify patients for personalised medicine. Thus, the molecular characterisation of tumours must enter a new era including functional studies of proteins such as endocytosis and membrane trafficking.

## Data Availability

Not applicable.

## Supplementary Materials

The following are available online at https://www.mdpi.com/article/10.3390/cells10113258/s1, Figure S1: Protein expression profile of the 3 GBM cell lines studied upon gefitinib treatment; Figure S2: GME of EGFR requires Rab5 activation.

## References

[1] An Z., Aksoy O., Zheng T., Fan Q.-W., Weiss W.A. (2018). Epidermal growth factor receptor (EGFR) and EGFRvIII in glioblastoma (GBM): Signaling pathways and targeted therapies. Oncogene.

[2] Eskilsson E., Røsland G.V., Solecki G., Wang Q., Harter P.N., Graziani G., Verhaak R.G.W., Winkler F., Bjerkvig R., Miletic H. (2018). EGFR heterogeneity and implications for therapeutic intervention in glioblastoma. Neuro-Oncol..

[3] Henriksen L., Grandal M.V., Knudsen S.L.J., van Deurs B., Grøvdal L.M. (2013). Internalization Mechanisms of the Epidermal Growth Factor Receptor after Activation with Different Ligands. PLoS ONE.

[4] Sigismund S., Argenzio E., Tosoni D., Cavallaro E., Polo S., Di Fiore P.P. (2008). Clathrin-Mediated Internalization Is Essential for Sustained EGFR Signaling but Dispensable for Degradation. Dev. Cell.

[5] Tomas A., Futter C.E., Eden E.R. (2014). EGF receptor trafficking: Consequences for signaling and cancer. Trends Cell Biol..

[6] Jovic M., Sharma M., Rahajeng J., Caplan S. (2010). The early endosome: A busy sorting station for proteins at the crossroads. Histol. Histopathol..

[7] Al-Akhrass H., Naves T., Vincent F., Magnaudeix A., Durand K., Bertin F., Melloni B., Jauberteau M.-O., Lalloué F. (2017). Sortilin limits EGFR signaling by promoting its internalization in lung cancer. Nat. Commun..

[8] Kondapalli K.C., Llongueras J.P., Capilla-González V., Prasad H., Hack A., Smith C., Guerrero-Cázares H., Quiñones-Hinojosa A., Rao R. (2015). A leak pathway for luminal protons in endosomes drives oncogenic signalling in glioblastoma. Nat. Commun..

[9] Walsh A.M., Kapoor G.S., Buonato J.M., Mathew L.K., Bi Y., Davuluri R.V., Martinez-Lage M., Simon M.C., O’Rourke D.M., Lazzara M.J. (2015). Sprouty2 Drives Drug Resistance and Proliferation in Glioblastoma. Mol. Cancer Res. MCR.

[10] Wang X., Wang Z., Zhang Y., Wang Y., Zhang H., Xie S., Xie P., Yu R., Zhou X. (2019). Golgi phosphoprotein 3 sensitizes the tumour suppression effect of gefitinib on gliomas. Cell Prolif..

[11] Ying H., Zheng H., Scott K., Wiedemeyer R., Yan H., Lim C., Huang J., Dhakal S., Ivanova E., Xiao Y. (2010). Mig-6 controls EGFR trafficking and suppresses gliomagenesis. Proc. Natl. Acad. Sci. USA.

[12] Grandal M.V., Zandi R., Pedersen M.W., Willumsen B.M., van Deurs B., Poulsen H.S. (2007). EGFRvIII escapes down-regulation due to impaired internalization and sorting to lysosomes. Carcinogenesis.

[13] Cao X., Zhu H., Ali-Osman F., Lo H.-W. (2011). EGFR and EGFRvIII undergo stress- and EGFR kinase inhibitor-induced mitochondrial translocalization: A potential mechanism of EGFR-driven antagonism of apoptosis. Mol. Cancer.

[14] Tan X., Lambert P.F., Rapraeger A.C., Anderson R.A. (2016). Stress-Induced EGFR Trafficking: Mechanisms, Functions, and Therapeutic Implications. Trends Cell Biol..

[15] Tan X., Thapa N., Sun Y., Anderson R.A. (2015). A Kinase-Independent Role for EGF Receptor in Autophagy Initiation. Cell.

[16] Tomas A., Vaughan S.O., Burgoyne T., Sorkin A., Hartley J.A., Hochhauser D., Futter C.E. (2015). WASH and Tsg101/ALIX-dependent diversion of stress-internalized EGFR from the canonical endocytic pathway. Nat. Commun..

[17] Dittmann K., Mayer C., Fehrenbacher B., Schaller M., Raju U., Milas L., Chen D.J., Kehlbach R., Rodemann H.P. (2005). Radiation-induced Epidermal Growth Factor Receptor Nuclear Import Is Linked to Activation of DNA-dependent Protein Kinase. J. Biol. Chem..

[18] Zwang Y., Yarden Y. (2006). p38 MAP kinase mediates stress-induced internalization of EGFR: Implications for cancer chemotherapy. EMBO J..

[19] Jones S., King P.J., Antonescu C.N., Sugiyama M.G., Bhamra A., Surinova S., Angelopoulos N., Kragh M., Pedersen M.W., Hartley J.A. (2020). Targeting of EGFR by a combination of antibodies mediates unconventional EGFR trafficking and degradation. Sci. Rep..

[20] Keir S.T., Chandramohan V., Hemphill C.D., Grandal M.M., Melander M.C., Pedersen M.W., Horak I.D., Kragh M., Desjardins A., Friedman H.S. (2018). Sym004-induced EGFR elimination is associated with profound anti-tumor activity in EGFRvIII patient-derived glioblastoma models. J. Neurooncol..

[21] Liao H.-J., Carpenter G. (2009). Cetuximab/C225-Induced Intracellular Trafficking of Epidermal Growth Factor Receptor. Cancer Res..

[22] Blandin A.-F., Cruz Da Silva E., Mercier M.-C., Glushonkov O., Didier P., Dedieu S., Schneider C., Devy J., Etienne-Selloum N., Dontenwill M. (2021). Gefitinib induces EGFR and α5β1 integrin co-endocytosis in glioblastoma cells. Cell. Mol. Life Sci..

[23] Coker E.A., Mitsopoulos C., Tym J.E., Komianou A., Kannas C., Di Micco P., Villasclaras Fernandez E., Ozer B., Antolin A.A., Workman P. (2019). canSAR: Update to the cancer translational research and drug discovery knowledgebase. Nucleic Acids Res..

[24] Perrot G., Langlois B., Devy J., Jeanne A., Verzeaux L., Almagro S., Sartelet H., Hachet C., Schneider C., Sick E. (2012). LRP-1--CD44, a new cell surface complex regulating tumor cell adhesion. Mol. Cell. Biol..

[25] Blandin A.-F., Noulet F., Renner G., Mercier M.-C., Choulier L., Vauchelles R., Ronde P., Carreiras F., Etienne-Selloum N., Vereb G. (2016). Glioma cell dispersion is driven by α5 integrin-mediated cell–matrix and cell–cell interactions. Cancer Lett..

[26] Sousa L.P., Lax I., Shen H., Ferguson S.M., De Camilli P., Schlessinger J. (2012). Suppression of EGFR endocytosis by dynamin depletion reveals that EGFR signaling occurs primarily at the plasma membrane. Proc. Natl. Acad. Sci. USA.

[27] Kirchhausen T., Macia E., Pelish H.E. (2008). Use of dynasore, the small molecule inhibitor of dynamin, in the regulation of endocytosis. Methods Enzymol..

[28] Robertson M.J., Deane F.M., Robinson P.J., McCluskey A. (2014). Synthesis of Dynole 34-2, Dynole 2-24 and Dyngo 4a for investigating dynamin GTPase. Nat. Protoc..

[29] Park R.J., Shen H., Liu L., Liu X., Ferguson S.M., Camilli P.D. (2013). Dynamin triple knockout cells reveal off target effects of commonly used dynamin inhibitors. J. Cell Sci..

[30] Barbieri M.A., Roberts R.L., Gumusboga A., Highfield H., Alvarez-Dominguez C., Wells A., Stahl P.D. (2000). Epidermal Growth Factor and Membrane Trafficking. J. Cell Biol..

[31] Chen P.-I., Kong C., Su X., Stahl P.D. (2009). Rab5 Isoforms Differentially Regulate the Trafficking and Degradation of Epidermal Growth Factor Receptors. J. Biol. Chem..

[32] Dinneen J.L., Ceresa B.P. (2004). Continual expression of Rab5(Q79L) causes a ligand-independent EGFR internalization and diminishes EGFR activity. Traffic Cph. Den..

[33] Ceresa B., Lotscher M., Schmid S. (2001). Receptor and Membrane Recycling Can Occur with Unaltered Efficiency Despite Dramatic Rab5(Q79L)-induced Changes in Endosome Geometry. J. Biol. Chem..

[34] Chen X., Wang Z. (2001). Regulation of epidermal growth factor receptor endocytosis by wortmannin through activation of Rab5 rather than inhibition of phosphatidylinositol 3-kinase. EMBO Rep..

[35] Etique N., Verzeaux L., Dedieu S., Emonard H. (2013). LRP-1: A checkpoint for the extracellular matrix proteolysis. BioMed. Res. Int..

[36] Bu G., Geuze H.J., Strous G.J., Schwartz A.L. (1995). 39 kDa receptor-associated protein is an ER resident protein and molecular chaperone for LDL receptor-related protein. EMBO J..

[37] Bu G., Schwartz A.L. (1998). RAP, a novel type of ER chaperone. Trends Cell Biol..

[38] Maritzen T., Schachtner H., Legler D.F. (2015). On the move: Endocytic trafficking in cell migration. Cell. Mol. Life Sci. CMLS.

[39] Wilson B.J., Allen J.L., Caswell P.T. (2018). Vesicle trafficking pathways that direct cell migration in 3D matrices and in vivo. Traffic Cph. Den..

[40] Díaz J., Mendoza P., Ortiz R., Díaz N., Leyton L., Stupack D., Quest A.F.G., Torres V.A. (2014). Rab5 is required in metastatic cancer cells for Caveolin-1-enhanced Rac1 activation, migration and invasion. J. Cell Sci..

[41] Eppinga R.D., Krueger E.W., Weller S.G., Zhang L., Cao H., McNiven M.A. (2012). Increased expression of the large GTPase dynamin 2 potentiates metastatic migration and invasion of pancreatic ductal carcinoma. Oncogene.

[42] Feng H., Liu K., Guo P., Zhang P., Cheng T., McNiven M., Johnson G., Hu B., Cheng S. (2012). Dynamin 2 Mediates PDGFRα-SHP-2-Promoted Glioblastoma Growth and Invasion. Oncogene.

[43] Razidlo G.L., Wang Y., Chen J., Krueger E.W., Billadeau D.D., McNiven M.A. (2013). Dynamin 2 Potentiates Invasive Migration of Pancreatic Tumor Cells through Stabilization of the Rac1 GEF Vav1. Dev. Cell.

[44] Yamada H., Takeda T., Michiue H., Abe T., Takei K. (2016). Actin bundling by dynamin 2 and cortactin is implicated in cell migration by stabilizing filopodia in human non-small cell lung carcinoma cells. Int. J. Oncol..

[45] Destaing O., Ferguson S.M., Grichine A., Oddou C., Camilli P.D., Albiges-Rizo C., Baron R. (2013). Essential Function of Dynamin in the Invasive Properties and Actin Architecture of v-Src Induced Podosomes/Invadosomes. PLoS ONE.

[46] Gong C., Zhang J., Zhang L., Wang Y., Ma H., Wu W., Cui J., Wang Y., Ren Z. (2015). Dynamin2 downregulation delays EGFR endocytic trafficking and promotes EGFR signaling and invasion in hepatocellular carcinoma. Am. J. Cancer Res..

[47] Khan I., Gril B., Steeg P.S. (2019). Metastasis Suppressors NME1 and NME2 Promote Dynamin 2 Oligomerization and Regulate Tumor Cell Endocytosis, Motility, and Metastasis. Cancer Res..

[48] Jian Z., Zhang L., Jin L., Lan W., Zhang W., Gao G. (2020). Rab5 regulates the proliferation, migration and invasion of glioma cells via cyclin E. Oncol. Lett..

[49] Zhou X., Xie S., Wu S., Qi Y., Wang Z., Zhang H., Lu D., Wang X., Dong Y., Liu G. (2017). Golgi phosphoprotein 3 promotes glioma progression via inhibiting Rab5-mediated endocytosis and degradation of epidermal growth factor receptor. Neuro-Oncology.

[50] Liu B., Su Y., Li T., Yuan W., Mo X., Li H., He Q., Ma D., Han W. (2015). CMTM7 knockdown increases tumorigenicity of human non-small cell lung cancer cells and EGFR-AKT signaling by reducing Rab5 activation. Oncotarget.

[51] Yuan W., Liu B., Wang X., Li T., Xue H., Mo X., Yang S., Ding S., Han W. (2017). CMTM3 decreases EGFR expression and EGF-mediated tumorigenicity by promoting Rab5 activity in gastric cancer. Cancer Lett..

[52] Ye C., Pan B., Xu H., Zhao Z., Shen J., Lu J., Yu R., Liu H. (2019). Co-Delivery of GOLPH3 siRNA and gefitinib by cationic lipid-PLGA nanoparticles improves EGFR-targeted therapy for glioma. J. Mol. Med. Berl. Ger..

[53] Cavalli V., Vilbois F., Corti M., Marcote M.J., Tamura K., Karin M., Arkinstall S., Gruenberg J. (2001). The stress-induced MAP kinase p38 regulates endocytic trafficking via the GDI:Rab5 complex. Mol. Cell.

[54] Macé G., Miaczynska M., Zerial M., Nebreda A.R. (2005). Phosphorylation of EEA1 by p38 MAP kinase regulates μ opioid receptor endocytosis. EMBO J..

[55] Peng K., Dai Q., Wei J., Shao G., Sun A., Yang W., Lin Q. (2016). Stress-induced endocytosis and degradation of epidermal growth factor receptor are two independent processes. Cancer Cell Int..

[56] Tomas A., Jones S., Vaughan S.O., Hochhauser D., Futter C.E. (2017). Stress-Specific p38 MAPK activation is sufficient to drive EGFR endocytosis but not its nuclear translocation. J. Cell Sci..

[57] Vergarajauregui S., Miguel A.S., Puertollano R. (2006). Activation of p38 Mitogen-Activated Protein Kinase Promotes Epidermal Growth Factor Receptor Internalization. Traffic Cph. Den..

[58] Tanaka T., Ozawa T., Oga E., Muraguchi A., Sakurai H. (2018). Cisplatin-induced non-canonical endocytosis of EGFR via p38 phosphorylation of the C-terminal region containing Ser-1015 in non-small cell lung cancer cells. Oncol. Lett..

[59] Soeda A., Lathia J., Williams B.J., Wu Q., Gallagher J., Androutsellis-Theotokis A., Giles A.J., Yang C., Zhuang Z., Gilbert M.R. (2017). The p38 signaling pathway mediates quiescence of glioma stem cells by regulating epidermal growth factor receptor trafficking. Oncotarget.

[60] Baldwin R.M., Garratt-Lalonde M., Parolin D.A.E., Krzyzanowski P.M., Andrade M.A., Lorimer I.A.J. (2006). Protection of glioblastoma cells from cisplatin cytotoxicity via protein kinase C ι -mediated attenuation of p38 MAP kinase signaling. Oncogene.

[61] Morello V., Cabodi S., Sigismund S., Camacho-Leal M.P., Repetto D., Volante M., Papotti M., Turco E., Defilippi P. (2011). β1 integrin controls EGFR signaling and tumorigenic properties of lung cancer cells. Oncogene.

[62] Theret L., Jeanne A., Langlois B., Hachet C., David M., Khrestchatisky M., Devy J., Hervé E., Almagro S., Dedieu S. (2017). Identification of LRP-1 as an endocytosis and recycling receptor for β1-integrin in thyroid cancer cells. Oncotarget.

[63] Boyé K., Pujol N., Alves I.D., Chen Y.-P., Daubon T., Lee Y.-Z., Dedieu S., Constantin M., Bello L., Rossi M. (2017). The role of CXCR3/LRP1 cross-talk in the invasion of primary brain tumors. Nat. Commun..

[64] Chew H.Y., De Lima P.O., Gonzalez Cruz J.L., Banushi B., Echejoh G., Hu L., Joseph S.R., Lum B., Rae J., O’Donnell J.S. (2020). Endocytosis Inhibition in Humans to Improve Responses to ADCC-Mediating Antibodies. Cell.

[65] Joseph S.R., Gaffney D., Barry R., Hu L., Banushi B., Wells J.W., Lambie D., Strutton G., Porceddu S.V., Burmeister B. (2019). An Ex Vivo Human Tumor Assay Shows Distinct Patterns of EGFR Trafficking in Squamous Cell Carcinoma Correlating to Therapeutic Outcomes. J. Investig. Dermatol..

