# Supplementary figures and images for: Role of Endocytosis Proteins in Gefitinib-Mediated EGFR Internalisation in Glioma Cells
Source: Cells. 2021 Nov 21;10(11):3258. doi: 10.3390/cells10113258 (PMC8618144; doi:10.3390/cells10113258)

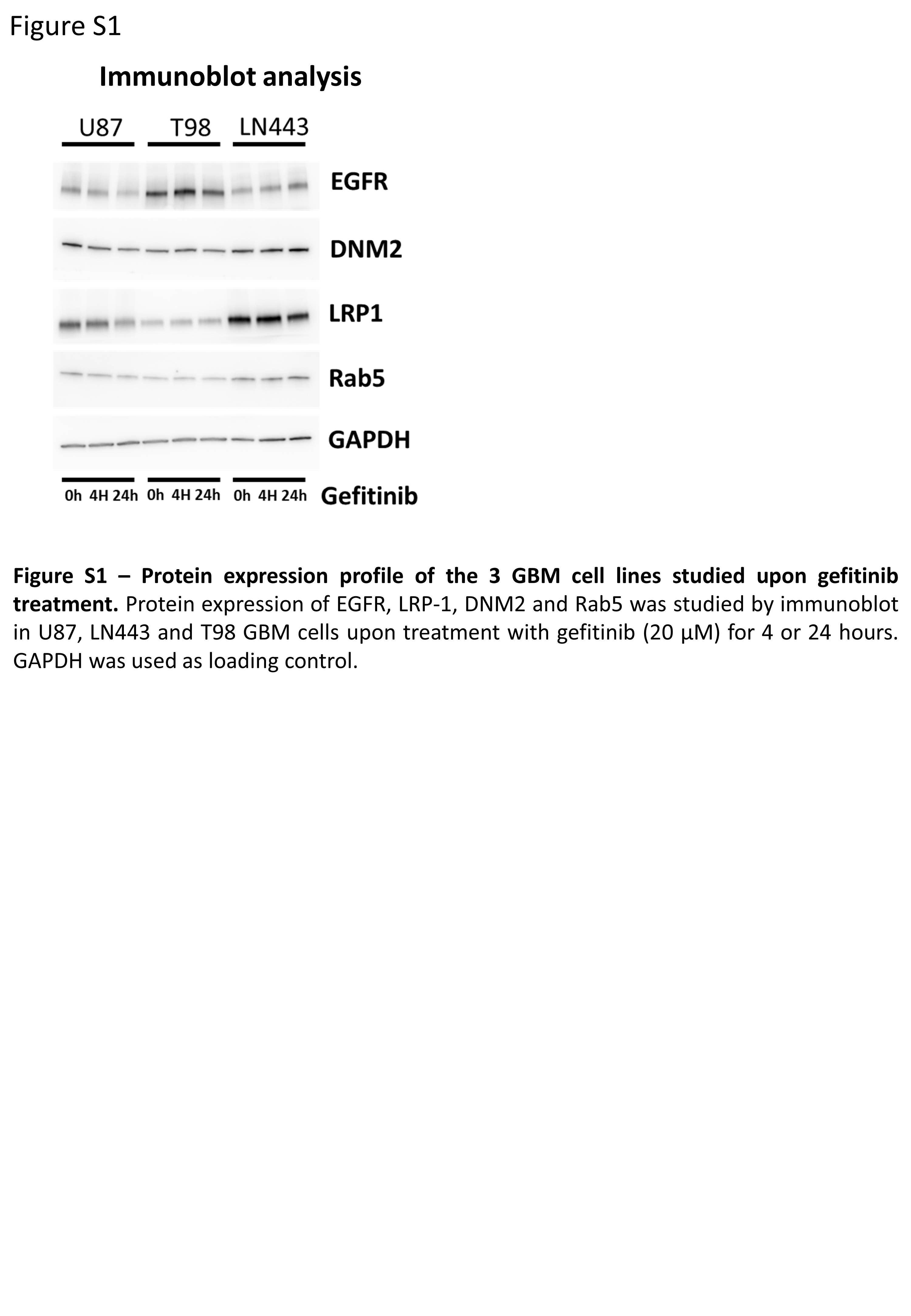

Supplement: Supplementary file 1 [file cells-10-03258-s001.zip › CruzdaSilva et coll_2021_Cells_SuppFig/Diapositive1.TIF]

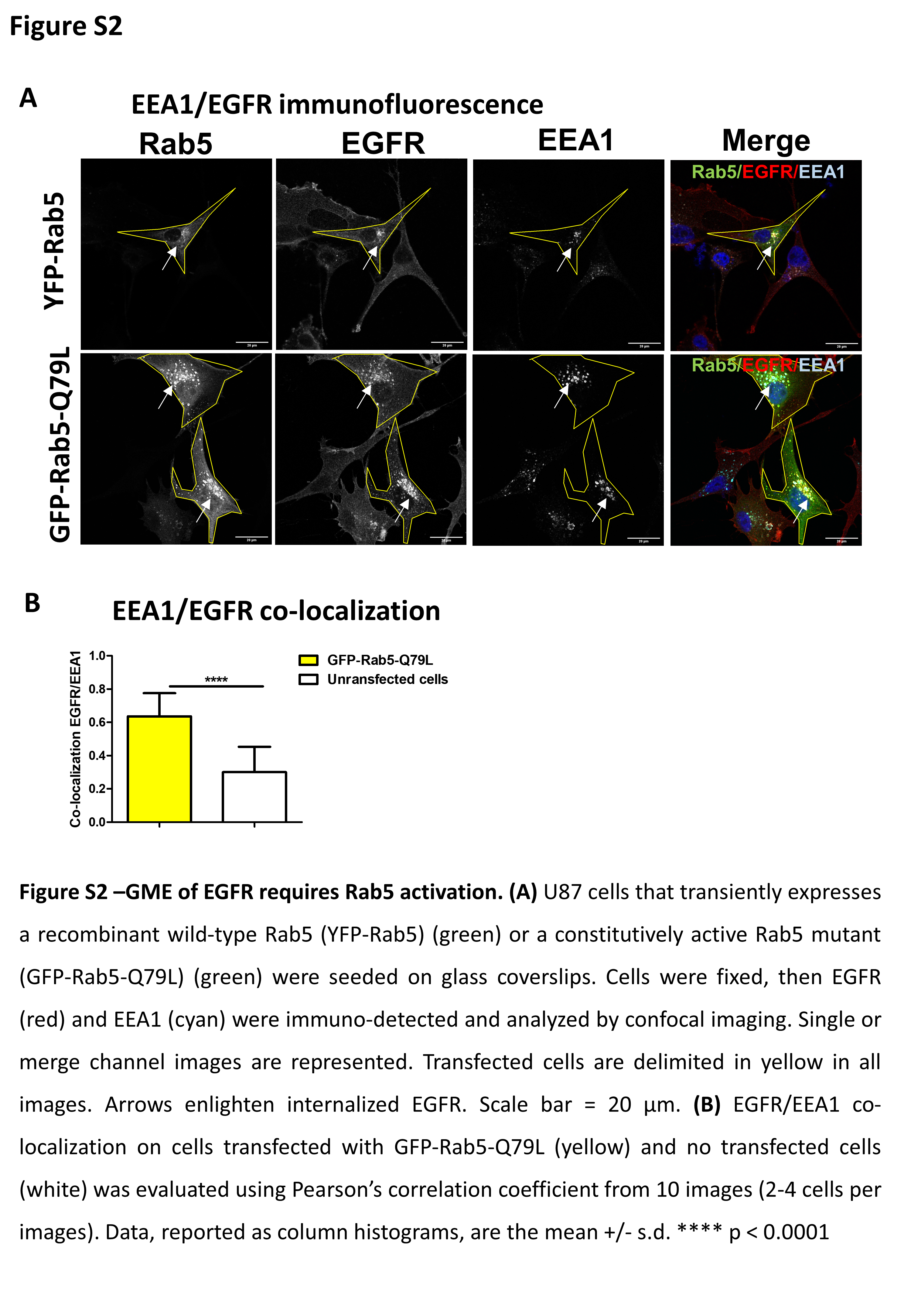

Supplement: Supplementary file 1 [file cells-10-03258-s001.zip › CruzdaSilva et coll_2021_Cells_SuppFig/Diapositive2.TIF]
